# Variability in prostate and seminal vesicle delineations defined on magnetic resonance images, a multi-observer, -center and -sequence study

**DOI:** 10.1186/1748-717X-8-126

**Published:** 2013-05-24

**Authors:** Tufve Nyholm, Joakim Jonsson, Karin Söderström, Per Bergström, Andreas Carlberg, Gunilla Frykholm, Claus F Behrens, Poul Flemming Geertsen, Redas Trepiakas, Scott Hanvey, Azmat Sadozye, Jawaher Ansari, Hazel McCallum, John Frew, Rhona McMenemin, Björn Zackrisson

**Affiliations:** 1 Department for Radiation Sciences, Radiation Physics, Umeå University, Umeå, Sweden; 2 Department for Radiation Sciences, Oncology, Umeå University, Umeå, Sweden; 3 Department of Medical Physics, Karolinska University Hospital, Solna, Sweden; 4 Department of Oncology, Karolinska University Hospital, Solna, Sweden; 5 Department of Oncology, Herlev University Hospital, Herlev, Denmark; 6 Department of Oncology, Hillerød Hospital, Hillerød, Denmark; 7 Department of Clinical Physics and Bioengineering, Radiotherapy Physics, Beatson West of Scotland Cancer Centre, Glasgow, Scotland, G12 0YN, UK; 8 Department of Clinical Oncology, Beatson West of Scotland Cancer Centre, Glasgow, Scotland, G12 0YN, UK; 9 Northern Centre for Cancer Care, Newcastle-Upon-Tyne Hospitals NHS Trust, High Heaton, Newcastle upon Tyne, UK

**Keywords:** Prostate, Seminal-vesicles, Delineation, Magnetic resonance imaging, Radiotherapy, Variability

## Abstract

**Background:**

The use of magnetic resonance (MR) imaging as a part of preparation for radiotherapy is increasing. For delineation of the prostate several publications have shown decreased delineation variability using MR compared to computed tomography (CT). The purpose of the present work was to investigate the intra- and inter-physician delineation variability for prostate and seminal vesicles, and to investigate the influence of different MR sequence settings used clinically at the five centers participating in the study.

**Methods:**

MR series from five centers, each providing five patients, were used. Two physicians from each center delineated the prostate and the seminal vesicles on each of the 25 image sets. The variability between the delineations was analyzed with respect to overall, intra- and inter-physician variability, and dependence between variability and origin of the MR images, i.e. the MR sequence used to acquire the data.

**Results:**

The intra-physician variability in different directions was between 1.3 - 1.9 mm and 3 – 4 mm for the prostate and seminal vesicles respectively (1 std). The inter-physician variability for different directions were between 0.7 – 1.7 mm and approximately equal for the prostate and seminal vesicles. Large differences in variability were observed for individual patients, and also for individual imaging sequences used at the different centers. There was however no indication of decreased variability with higher field strength.

**Conclusion:**

The overall delineation variability is larger for the seminal vesicles compared to the prostate, due to a larger intra-physician variability. The imaging sequence appears to have a large influence on the variability, even for different variants of the T2-weighted spin-echo based sequences, which were used by all centers in the study.

## Introduction

Successful radiotherapy depends on high geometric and dosimetric accuracy and precision. The introduction of treatment planning and dose calculation in 3D, more than two decades ago, has provided the clinicians with very good control over the dosimetric aspects of the treatment with typical relative errors in the order of a few percent. The more recent introduction of intensity modulated radiotherapy (IMRT) [1] has made it possible to shape the dose distribution to closely match the target volume and the use of image guided radiotherapy (IGRT) [2] enables reproducible patient positioning at every treatment fraction. At present, we have come close to a point where we can “hit the target” with the right dose every time with minimal dose deposition outside the intended volume. Hence, treatment precision has dramatically improved. However, there are still problems to be solved, as described by Njeh [3]; the uncertainty in the definition of the target. Sharp dose gradients are more a hazard than a benefit, if the geometric uncertainty in delineation is large.

The use of magnetic resonance (MR) imaging alone, or together with computed tomography (CT), improves the target delineation accuracy for many diagnoses [4,5] and MR imaging is today in routine clinical use at many centers as a part of the preparation for radiotherapy. The dedicated MR examination for radiotherapy treatment planning involves issues not present in the diagnostic setting. The patient should ideally be imaged in the same position as during treatment, including fixations [6] which influence both the coil setup and image quality [7,8]. The geometric accuracy of the images is crucial which increase demands on the choice of sequences and bandwidth [9] and the sequences and image planes should be optimized for determination of the precise geometrical extent of an already known pathology.

There are two alternative ways of incorporating the MR into the radiotherapy workflow; either the MR images are seen as a complement to the CT for target definition or the MR replaces the CT throughout the entire treatment process. The CT/MR workflow is already established in many centers, but suffers from drawbacks in terms of increased workload and potential introduction of geometric errors resulting from the image registration procedure [10,11]. Fully MR based workflows have been described in the literature [12-15] and are considered feasible.

For prostate cancer patients, the use of MR alone or in combination with CT has been shown to reduce inter-observer variability in target definition and reduce the treatment volume [16-19]. The treatment of prostate cancer has been considered one of the most straightforward diagnoses for an MR only workflow, as the dose calculation accuracy in the pelvic region is adequate with bulk density assignments [20,21] and the commonly used gold markers are visible with reliable geometric accuracy [22]. In addition to the technical challenges with the MR based workflow, one must also consider that the physicians need to adapt to a target definition process without CT information, and that the MR sequences need to be optimized for target definition purposes.

The aim of the present multi-center study is to evaluate the intra- and inter-physician variability of prostate and seminal vesicle volume delineations based on MR sequences from five different radiotherapy centers in the clinical setting. All centers participating in the study were at the time investigating the use of an MR based workflow for the treatment of prostate cancer. As part of this process it was considered important to perform an inter-clinic comparison of both the standard clinical MR images and the interpretation of the images by the physicians. The observed variations can be assumed to reflect the clinical reality as the images were acquired with the standard clinical protocol and the physicians were instructed to perform the delineation as for an ordinary clinical case.

## Methods and materials

Five centers were involved in the study; Umeå University Hospital (Umeå, Sweden), Karolinska Hospital (Stockholm, Sweden), Herlev Hospital (Copenhagen, Denmark), Newcastle Upon Tyne Hospitals NHS Trust (Newcastle, United Kingdom) and Beatson West of Scotland Cancer Centre (Glasgow, United Kingdom). All centers were, at the time of the study, routinely using MRI data in their clinical practice for target definition for prostate cancer patients, except Karolinska who was in the startup process. Both participating physicians from Karolinska did however have extensive previous experience (>5 years) of prostate delineations on MR images from other hospitals. The different scanners and sequences used in the study are listed in Table 1. All centers had chosen to use spin-echo based T2 weighted images as primary bases for target delineation.

### Imaging and preparation of data

Five consecutive patients scheduled for radiotherapy of the prostate were selected from each site. All patients had MR examinations as part of their standard preparation for radiotherapy. The axial images which were typically used for target delineation were anonymized and sent to the study coordinator. The 5 image series from each of the 5 sites were tagged as CT studies in the DICOM files to enable delineations to be performed directly on the MR data in all oncology delineation software applications. The set of 25 image series were then sent to each site and imported into the clinically used treatment planning systems or dedicated delineation software.

### Delineations

Two physicians from each site independently delineated the prostate volume and the seminal vesicles. The instruction was: “*Both prostate and vesicles should be delineated as if a clinical case with high risk for vesicle involvement*”. The prostate and the vesicle delineations were stored as separate structure sets. After finalizing the delineations, the structure sets were returned to the study coordinator as DICOM RTstruct files for analysis.

### Analysis

The total dataset consisted of 25 patients, with delineations from 10 physicians for each patient. All structures for an individual patient were defined in the same coordinate system and could be directly compared. The prostate and vesicle volumes were analyzed separately.

**Table 1:** The sequence used at center C were a 3D sequence (Siemens, SPACE), while the other clinics used 2D sequences

**Center**	**Delienation software**	**Scanner**	**Field strength**	**Echo time (ms)**	**Rep. time (ms)**	**Slice thickness (mm)**	**Pixel size (mm**^**2**^**)**
A	Eclipse	Philips Panorama	1.0 T	110	4471	2	0.91 × 0.91
B	Eclipse	Siemens Verio	3 T	92	3440	3.6	0.52 × 0.52
C	ProSoma[MedCom]	Siemens Espree	1.5 T	125	3000	1.7	0.78 × 0.78
D	Oncentra	Siemens Espree	1.5 T	115	10200	3.3	1.17 × 1.17
E	Eclipse	GE Signa HDxt	1.5 T	90	2520	2.5	0.82 × 0.82

**Figure 1:**
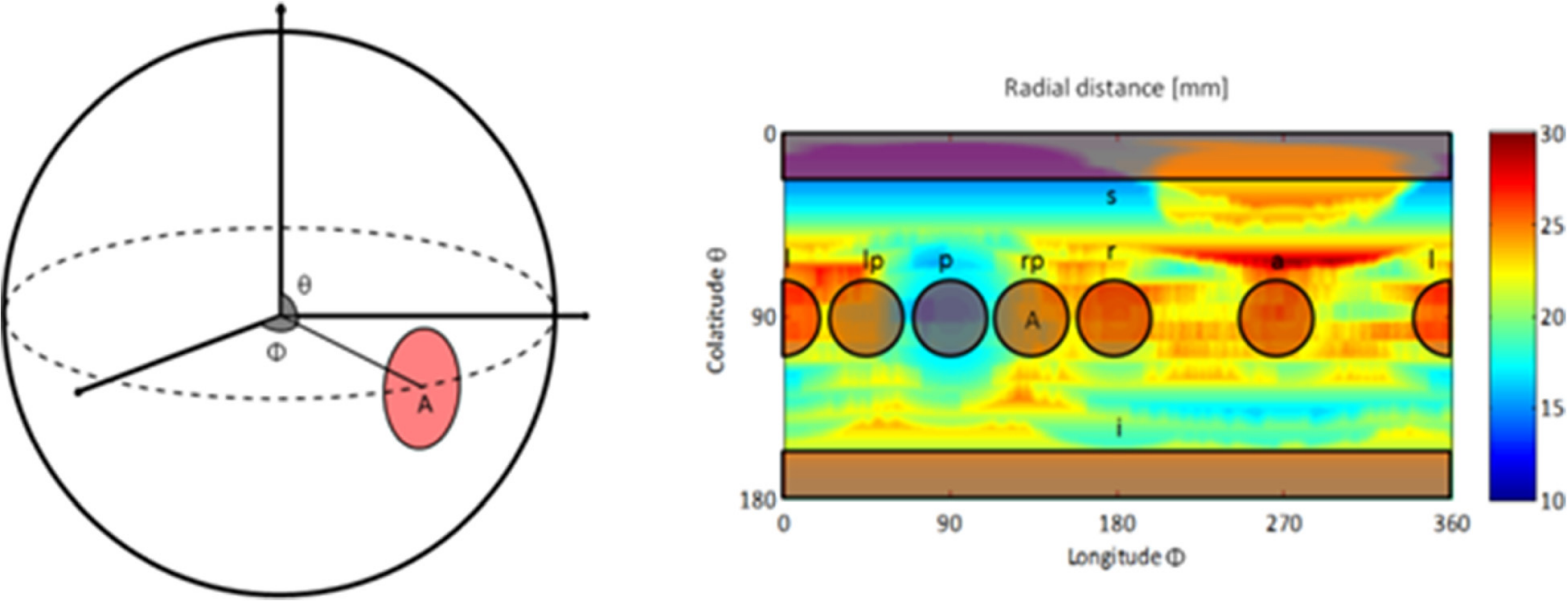
**The radial distances from the center of mass to each delineation is a function of the angles *θ *and ∅.** The distance in different directions for an individual delineation was calculated as the average distance over a small solid angle in each direction, (left (l), left-posterior (lp), posterior (p), right-posterior (rp), right (r), anterior (a), superior (s), inferior (i)). The area A, corresponding to the right-posterior direction, illustrates the relation between the 3D and 2D representation. The example in the figure is based on one delineation of one prostate.

For the prostate, the first step was to calculate the joint center of mass for all delineations for each patient. The distance from the center was calculated for each delineation in the directions right, left, anterior, posterior, superior, inferior, right-posterior and left-posterior. To reduce the influence of small scale variations in the structure sets and create a representative measure for the distance, the average over a solid angle Ω =0.49 sr was used (Figure 1). This procedure provides a single numerical measure for the distance in the different directions for each patient and delineation.

The joint center of mass for each patient was also used as a starting point for the analysis of the vesicle delineations. The shape of the vesicles does not, however, allow the same analysis approach due to the sometimes concave surface. Instead, the maximum distance in the right, left, anterior, posterior, superior and inferior directions from the center of mass were calculated for each delineation.

### Nomenclature

A specific physician is denoted q  and a specific patient p. The complete set of physicians is denoted Q  and a set of patients is denoted P. The center from which a specific images originates is called imaging center (IC), and the center where a specific delineation is performed is called delineation center (DC). $x_{p,q}^{\mathrm{var}}$ is the observed variable var, which can be either the volume or the distance in a specific direction. The current work includes 25 patients (*N*_P_ = 25) and 10 physicians ( *N*_Q_ = 10). The average measure of each delineation characteristic was used as a golden standard, and was calculated as

(1) $${\bar{x}}_{p,*}^{\mathrm{var}}=\frac{1}{N_{Q}}\sum_{q\in Q}x_{p,q}^{\mathrm{var}}$$

To remove the systematic variations connected to specific patients we formed the variable

(2) $$y_{p,q}^{\mathrm{var}}=x_{p,q}^{\mathrm{var}}-{\bar{x}}_{p,*}^{\mathrm{var}}$$

i.e. the difference between an individual physician (q) delineation on a specific patient (p) and the average delineation over all physicians, for the delineation characteristic var.

To refer to a specific subgroup of patients imaged at a specific IC, we use the notation p&z.epsi;IC. To refer to the subgroup of physicians belonging to a specific DC we

 use the notation q&z.epsi;DC. In the result section we use the notation *A*[*variable*]_parameter_ for the average, and *S*[*variable*]_parameter_ for the standard deviation, where the parameter defines the group. For example $A{\left[{\bar{x}}_{p,*}^{\mathrm{var}}\right]}_{p\mathrm{\&z.epsi;}\mathrm{IC}}$ refers to the average measure of the delineation property var for all patients coming from imaging center IC.

### Statistical analysis

The normality of the data was checked through visual inspection of Q-Q plots. Most reported significant differences use a Bonferroni corrected 0.01 confidence level. The reason for the use of the strict significance levels was that the main purpose of the tests was to highlight the most pronounced effects in the dataset, where most factors can be expected to have influence. Two sided F-tests were used to compare distributions and t-tests to compare averages, unless otherwise indicated.

### Intra and inter-observer variation

**Figure 2:**
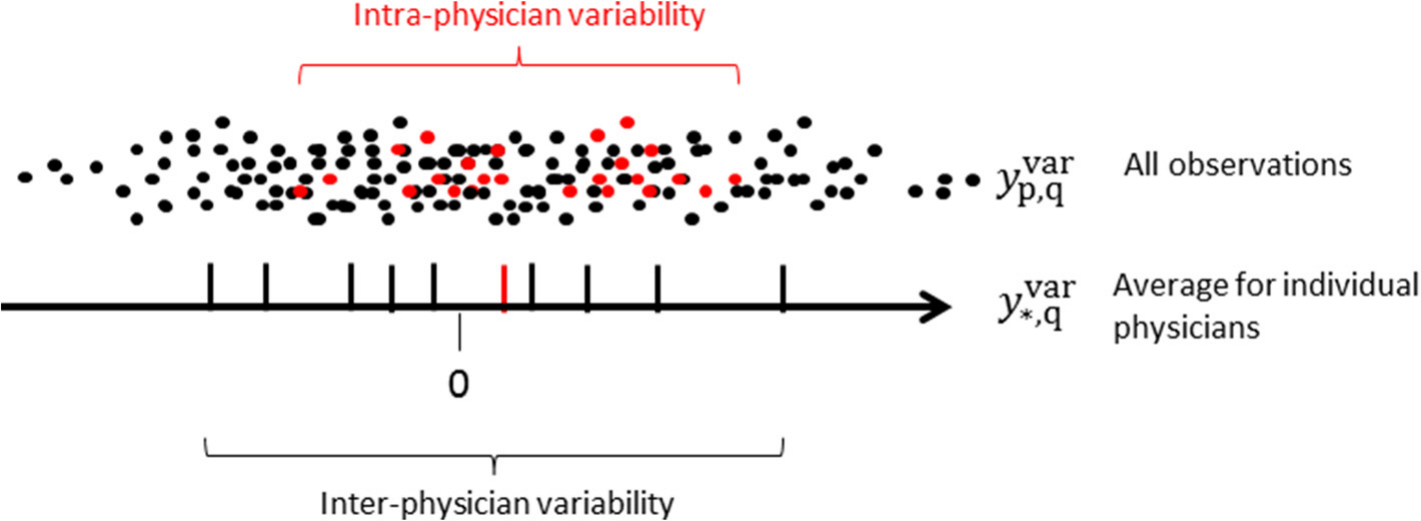
**Illustration of the separation of the overall variability into inter- and intra-physician components.** The red dots represent observations by an individual physician for the 25 patients. The red line indicate the average observation for the physician.

It was assumed that the variable $y_{p,q}^{\mathrm{var}}$ is dependent on physician (q) and patient (p), i.e.

(3) $$y_{p,q}^{\mathrm{var}}=w_{q}^{\mathrm{var}}+z_{p,q}^{\mathrm{var}}$$

where *w*_q_ is a factor that is only dependent on the physician, with expectation value $E{\left[w_{q}^{\mathrm{var}}\right]}_{q\in Q}\equiv 0$ and standard deviation $\sigma_{w}^{\mathrm{var}}$. $z_{p,q}^{\mathrm{var}}$ is a factor dependent on both patient and physician also with expectation value $E{\left[z_{p,q}^{\mathrm{var}}\right]}_{p\in P,q\in Q}\equiv 0$, and standard deviation $\sigma_{z}^{\mathrm{var}}$. Equation 3 is an ordinary one-way random effect Anova model [23], where $w_{q}^{\mathrm{var}}$ is the effect of the physician, hence $\sigma_{w}^{\mathrm{var}}$ is interpreted as the inter-physician variation, and $z_{p,q}^{\mathrm{var}}$ accounts for the residual variation, hence $\sigma_{z}^{\mathrm{var}}$ is interpreted as the intra-physician variation (Figure 2).

Estimates for the variances, i.e. ${\left(s_{z}^{\mathrm{var}}\right)}^{2}$ and ${\left(s_{w}^{\mathrm{var}}\right)}^{2}$, can be calculated using the equations

(4) $${\left(s_{z}^{\mathrm{var}}\right)}^{2}=\frac{1}{\left(N_{Q}-1\right)\left(N_{P}-1\right)}\sum_{p=1}^{N_{P}}\sum_{q=1}^{N_{Q}}{\left(y_{p,q}^{\mathrm{var}}-{\bar{y}}_{*,q}^{\mathrm{var}}\right)}^{2}$$

(5) $${\left(s_{z}^{\mathrm{var}}\right)}^{2}+N_{P}{\left(s_{w}^{\mathrm{var}}\right)}^{2}=\frac{N_{P}}{N_{Q}-1}\sum_{q=1}^{N_{Q}}{\left({\bar{y}}_{*,q}^{\mathrm{var}}\right)}^{2}$$

Confidence intervals for the true variabilities were found using simulation. The simulation was performed in a custom written Matlab™ Monte Carlo script. The script searched the $\sigma_{z}^{\mathrm{var}}$ and $\sigma_{w}^{\mathrm{var}}$ space to identify the area where the probability to get the observed $s_{z}^{\mathrm{var}}$ and $s_{w}^{\mathrm{var}}$ or more extreme values was below 5%.

## Results

### Normality

The differences between delineations from individual physicians and the average, i.e. $y_{p,q}^{\mathrm{var}}$, were approximately normally distributed for all scored variables. There was however a tendency that the largest deviations were larger than predicted with a Gaussian model, especially pronounced for the posterior, right-posterior and left-posterior directions for the prostate and for the volume and anterior direction for the vesicles.

### Delineation summary

**Figure 3:**
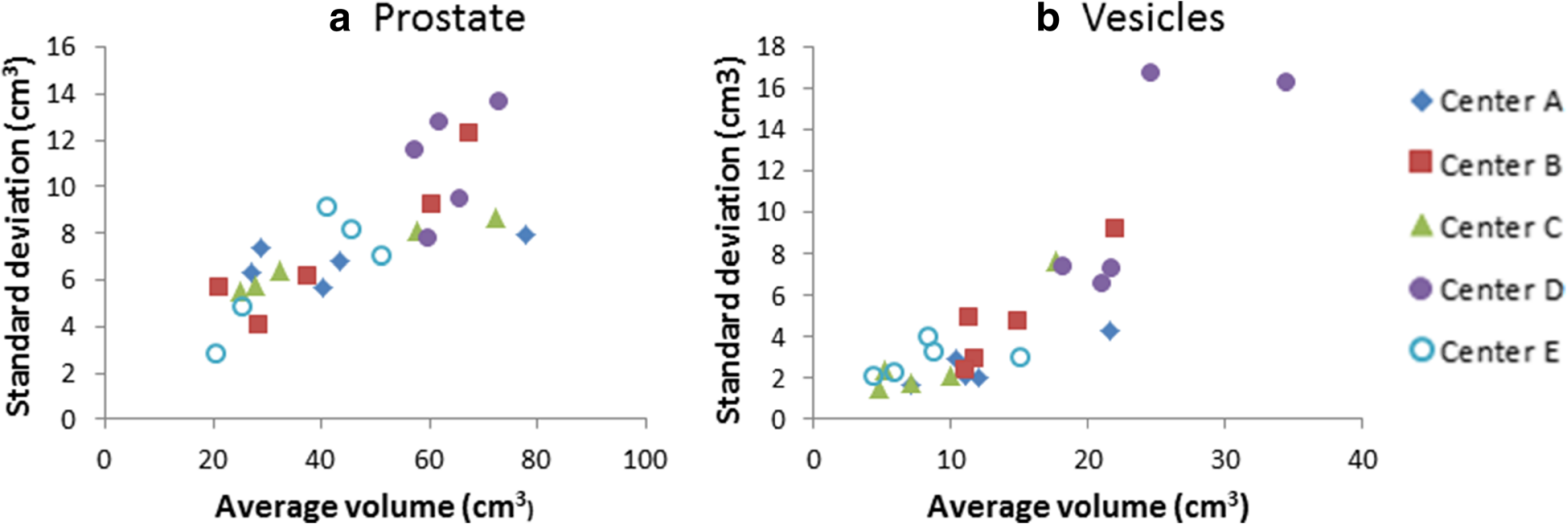
**The standard deviation,**$S{\left[x_{p,q}^{\mathrm{volume}}\right]}_{q},$**for the volume of the delineations for all 25 patients (prostate and vesicles), as function of the average volume,**${\left[x_{p,q}^{\mathrm{volume}}\right]}_{q}$**.** Each dot represents a patient. The imaging center is indicated by the dot shape.

As seen in Table 2 and Figure 3 there are apparent differences between the patient samples from the centers. The average volume, i.e. $A{\left[{\bar{x}}_{p,*}^{\mathrm{volume}}\right]}_{p\epsilon \mathrm{IC}}$, differed significantly both for the prostate and vesicles for patients with imaging center D compared to the others. The mean relative standard deviation, i.e. $A{\left[\frac{S{\left[x_{p,q}^{\mathrm{volume}}\right]}_{q\epsilon Q}}{{\bar{x}}_{p,*}^{\mathrm{var}}}\right]}_{p\epsilon \mathrm{IC}}$, was

**Table 2:** Average delineated prostate and vesicle volumes for patients from the different centers, and the mean relative standard deviations between the physicians

	**Prostate**		**Vesicles**	
**Imaging center (IC)**	**Average volume (cm**^**3**^**)**	**Mean relative standard deviation**	**Average volume (cm**^**3**^**)**	**Mean relative standard deviation**
**A**	44	18%	12	22%
**B**	43	18%	14	33%
**C**	43	18%	9	33%
**D**	63^(1)^	17%	24^(1)^	44%
**E**	37	17%	9	37%

(1) – Statistically different from the rest (p < 0.01 bonferroni corrected) 2-sided *t*-test.

 approximately the same for the different imaging centers (Table 2).

### Variability for different patients

The variability among the physicians differed for different patients, as can be seen in Tables 3 and 4 giving the median, max and min of the standard deviation for individual patients, i.e. $\max;\min;\mathrm{median}{\left[S{\left[y_{p,q}^{\mathrm{var}}\right]}_{q}\right]}_{p}$.

For the prostate, the highest frequency of large deviations ($y_{p,q}^{\mathrm{var}}>4\;\mathrm{mm})$ was found in the inferior and anterior directions (8%) each, followed by the superior direction (6%). The lowest frequency was found in the right, left and posterior directions (below 3%) while the frequency was around 5% in posterior-left and posterior-right directions.

For the vesicles the variability was larger as can be seen by comparing Tables 3 and 4. The highest frequency of large deviations $\left(y_{p,q}^{\mathrm{var}}>8\;\mathrm{mm}\right)$ was found in the right and left directions (6%), while the frequency was around 2% in the other directions.

### Physician variability

The influence of the delineating physician was large and significant for all investigated variables (p < 0.01, Kruskal-Wallis test, SPSS). Tables 5 and 6 gives the intra- and inter-physician variability (1 std) for the different delineation variables, for the prostate and the seminal vesicles, together with the 95% confidence interval for the true variability.

### Variability for different Sequences

**Figure 4:**
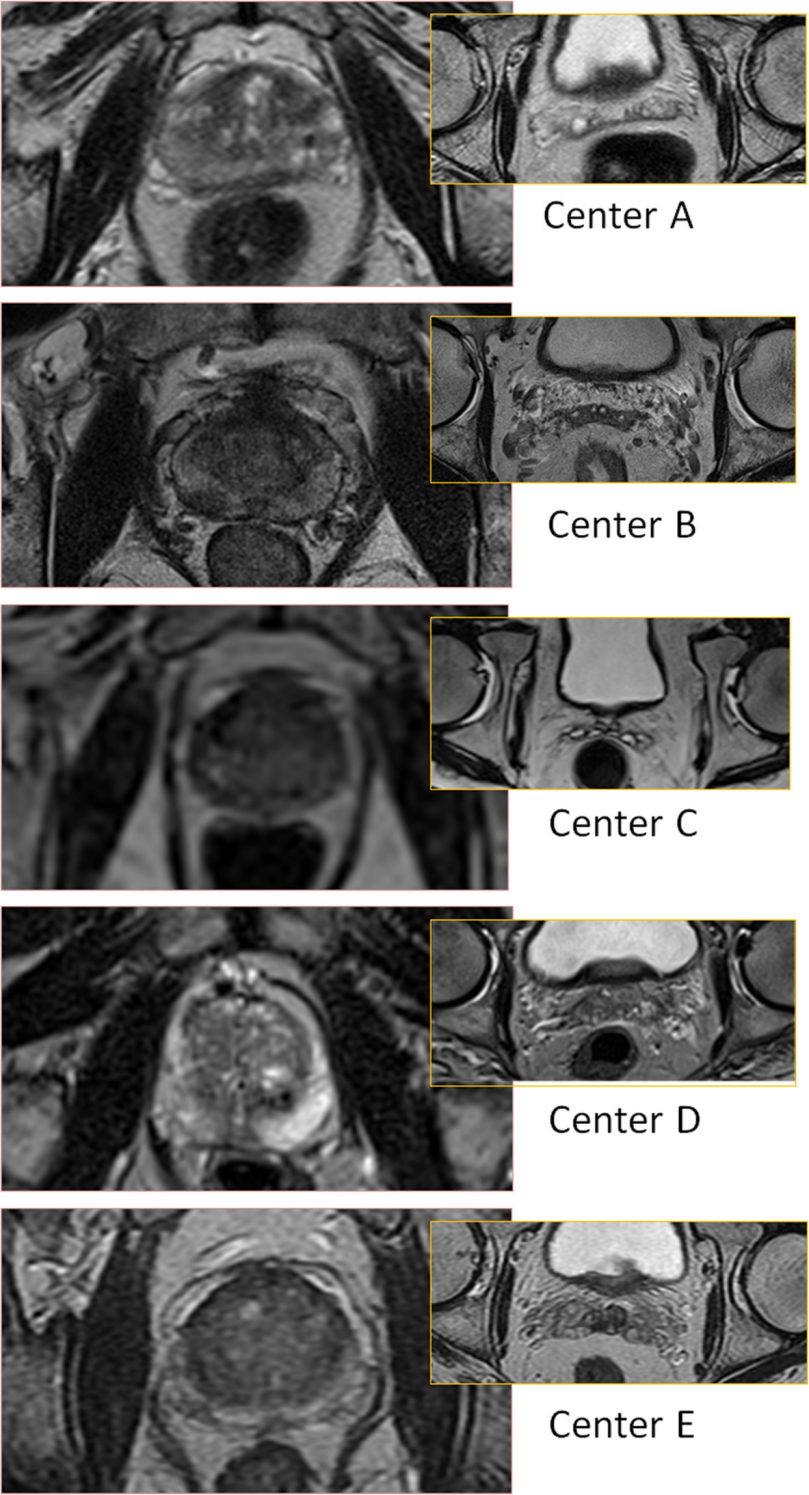
Examples of the image quality of the images from the different centers (prostate and seminal vesicles).

All centers participating in the study used T2 weighted images for target delineation. There were, however, noticeable differences in the image contrast, as can be appreciated in Figure 4. Tables 7 and 8 gives the variability scored for different imaging centers, i.e. $S{\left[y_{p,q}^{\mathrm{var}}\right]}_{p\in \mathrm{IC},q\in Q}$, together with the variability for physicians delineating on images from their home center, i.e. $S{\left[y_{p,q}^{\mathrm{var}}\right]}_{\left\{p,q\left|\mathrm{IC}=\mathrm{DC}\right)\right\}}$, and images from the other (foreign) centers, i.e. $S{\left[y_{p,q}^{\mathrm{var}}\right]}_{\left\{p,q\left|\mathrm{IC}\neq \mathrm{DC}\right)\right\}}$ .

The variability was in general smaller for home center delineations compared to foreign center delineations.

**Table 3:** The median, maximum and minimum observed variability for prostate delineation for an individual patient (1 std)

	**Anterior**	**Posterior**	**RightPost**	**Right**	**LeftPost**	**Left**	**Superior**	**Inferior**	**Volume**
	**(mm)**	**(mm)**	**(mm)**	**(mm)**	**(mm)**	**(mm)**	**(mm)**	**(mm)**	**(cm**^**3**^**)**
***Median***	1.9	0.8	1.5	1.4	1.7	1.4	1.8	2.5	7.4
***Max***	4.4	4.6	4.0	3.3	4.0	2.8	4.0	4.0	13.6
***Min***	0.9	0.4	0.9	0.9	0.7	0.6	1.1	1.0	2.8

 This was the pattern for all scored variables for the prostate, and was significant for posterior and right/posterior directions (2-sided F-test p < 0.01 Bonferroni corrected). For the vesicles the pattern was similar. The variability was significantly lower for the right and left directions with images from the physicians home center compared to foreign center (2-sided F-test p < 0.01 Bonferroni corrected).

## Discussion

Delineation errors have a direct effect on the quality of the treatment. An excessive target volume entails unnecessary risk of complications, while an undersized target reduces the chance of cure. The relationship between the target definition variability and the extent of the optimal margin to compensate for geometrical uncertainties is not completely clear. From a local control perspective the target definition variability should be considered a systematic uncertainty affecting the entire treatment, and should therefore be reflected in the employed margins. However, the uncertainty in the delineation is heavily dependent on both physician (Tables 5 and 6) and patient (Tables 3 and 4), which makes it inadequate to employ generalized margins to account for the variability. The opinion of the authors of the present work is that the responsibility to account for the delineation uncertainty should be placed on the physicians. The target volume should be delineated to cover the volume that the physician wants to treat; actively including volumes that are of benefit for the patient taking both local control probability and risk for side effects into account, and actively excluding volumes that for example are close to sensitive healthy tissues and the probability for tumorous growth is considered small. When only one physician delineates the target, the delineation from that physician is the best available estimate for the correctly defined target volume and should therefore be used without any additional generic margin accounting for the variability. Hence, the primary effect of improved imaging leading to decreased variability will not be a general possibility to reduce the standard margins, but will rather be reflected in a more uniform and generally increased treatment quality. Improved consistency will, as a secondary effect, improve the statistical power when evaluating and optimizing treatment protocols in clinical trials.

A way to decrease variability is through training and experience [24]. In Tables 7 and 8 it can be seen that physicians’ delineation on images from their home center generally were closer to the average compared to when delineating on images from foreign centers. In some directions the difference was up to 40% (for example posterior direction for the prostate). This effect may be attributed to customization and experience of the local MR sequence. Another way to potentially decrease the variability is to optimize the MR sequence. It is however ambitious to optimize with respect to the delineation variability. Data from the present work does not indicate reduced variability when using a 3 T scanner (center B), but the observations for the single 3 T scanner and only one sequence may not to be representative.

Intra-observer variation has often been reported based on repeated delineation on a single image set by one or several physicians. The approach used in this work is based on the analysis of variance theory [23] and has been described for the present application by Remeijer et al. [25]. It does not rely on multiple delineations by the same physician on individual images. To be able to make the separation we assumed that the intra-physician variability was equal for all physicians. In practice this was not the case. For all scored variables there was at least one physician with significantly different variability (F-test p < 0.01 Bonferroni corrected). In addition,

**Table 4:** The median, maximum and minimum observed variability for seminal vesicle delineation for an individual patient (1 std)

	**Anterior**	**Posterior**	**Right**	**Left**	**Superior**	**Inferior**	**Volume**
	**(mm)**	**(mm)**	**(mm)**	**(mm)**	**(mm)**	**(mm)**	**(cm**^**3**^**)**
***Median***	3.2	2.0	2.9	3.7	2.8	3.3	3.0
***Max***	7.2	7.5	8.5	7.6	5.8	8.0	16.8
***Min***	1.5	0.8	1.3	0.7	1.3	1.5	1.5

**Table 5:** Separation between intra- and inter-physician variability (1 std) for the prostate in different directions and for the volume

	**Anterior**	**Posterior**	**RightPost**	**Right**	**LeftPost**	**Left**	**Superior**	**Inferior**	**Volume**
	**(mm)**	**(mm)**	**(mm)**	**(mm)**	**(mm)**	**(mm)**	**(mm)**	**(mm)**	**(cm**^**3**^**)**
Intra	1.7	1.4	1.6	1.4	1.6	1.3	1.5	2.0	5.5
	(1.9-1.6)	(1.5-1.3)	(1.7-1.4)	(1.6-1.3)	(1.8-1.5)	(1.5-1.2)	(1.6-1.3)	(2.2-1.8)	(6.1-5.0)
Inter	1.5	0.7	1.3	1.0	1.4	0.9	1.6	1.7	6.03
	(2.8-1.0)	(1.3-0.4)	(2.4-0.9)	(1.75-0.6)	(2.5-0.9)	(1.7-0.6)	(2.9-1.1)	(3.1-1.1)	(11.0-4.0)

The numbers within brackets below each estimate indicates the 95% confidence interval.

 Tables 7 and 8 shows that the variability depends on the origin of the images (home vs. foreign center) as mentioned above. This phenomenon was not accounted for in the separation model. The numbers in Tables 5 and 6 are representative values describing the observations in the present study, but should be interpreted with these reservations in mind.

A concern when setting up the study was that the observed overall variability would primarily reflect the use of different clinical routines and traditions at the different centers. The separation of the variability into inter- and intra-physician components did however reveal that the intra-physician variability was dominating both for the prostate and especially for the seminal vesicles. There were significant differences between different delineation centers, the physicians from center A and D did on average delineate 20-30% larger prostate volumes compared to the physicians from centers B, C and E, but the dominating source for variability in individual directions was still the intra-physician variability. The increase of overall variability for the seminal vesicles delineation compared to the prostate delineation could be fully attributed to the larger intra-physician variability.

The inter-physician variability observed in the present study, summarized in Table 5, is approximately in line with the observations described in the literature. Rasch et al. found an inter-physician variability in the inferior region (apex) and superior region of around 1 mm (1.7 mm and 1.6 mm in present study) using axial MR images for 18 patients and with 3 observers [16]. The intra-observer variability was around 3 mm in both regions (2.0 mm and 1.5 mm in present study). It should be noted that Rasch et al. used a similar separation of variance as utilized in the present work, but the low number of physicians make the estimates for the inter-physician variability uncertain. Smith et al. reported inter-observer volume variability of 4.6 cm^3^ (6.1 cm^3^ in present study), and intra-physician volume variability of 2.7 cm^3^ based on repeated observations on same patient (5.1 cm^3^ in present study), in a study with 10 patients and 7 observers [26]. The large difference between the intra-physician variability in the present study compared to the study by Smith et al. could be due to the use of repeated delineations on the same image to estimate the intra-physician variability compared to separation of variances.

The results from the present study are also approximately in line with variability reported in the literature for CT based delineations. Fiorino et al. [27] has reported a study using 6 patients and 5 observers and found a short term intra-physician variation of 0.8, 1.1, 1.5 mm for the posterior, anterior and right/left directions to be compared with intra-physician variability 1.4, 1.7 resp. 1.9 mm found in the present study. The inter-observer standard deviation was estimated by Fiorino et al. to 1.4, 1.5 and 2.0 mm in the anterior, posterior and left-right directions, which should be compared with the approximately equivalent numbers found in Table 5, i.e. 0.7, 1.5 and 1.3 mm. For the vesicles Fiorino et al. reported inter-observer variability of 1.5, 2.8 and 2.3 mm in the posterior, anterior and lateral directions (1.0, 1.6, 1.5 mm in present work), and intra-observer variability of 1.2, 1.2, 1.5 (2.9, 3.9, 5.2 mm present work). The

**Table 6:** Separation between intra- and inter-physician variability (1 std) for the seminal vesicles

	**Anterior**	**Posterior**	**Right**	**Left**	**Superior**	**Inferior**	**Volume**
	**(mm)**	**(mm)**	**(mm)**	**(mm)**	**(mm)**	**(mm)**	**(cm**^**3**^**)**
Intra	3.9	2.9	3.6	3.8	2.9	3.5	5.7
	(4.3-3.6)	(3.2-2.6)	(4.0-3.3)	(4.2-3.5)	(3.2-2.7)	(3.8-3.1)	(5.2-6.3)
Inter	1.6	1.0	0.8	1.3	1.6	1.7	2.8
	(3.1-0.9)	(2.1-0.6)	(1.8-0.2)	(2.7-0.7)	(3.0-1.0)	(3.2-1.0)	(5.4-1.7)

The numbers in hard brackets for the right directions are calculated with one extreme outlier removed (>4 cm). The numbers within brackets below each estimate indicates the 95% confidence interval.

 comparison with Fiorino et al. does not reveal any substantial decrease in the variability when using MR compared to CT. This indicates that the benefit of MR is more in terms of accuracy than precision. To enable comparison with the results form Fiorino et al. in the right-left direction, the variability in the right and left directions from the present work was added together assuming these are independent variables.

The present work is based on a total of 250 delineations performed by 10 physicians on 25 patients. This is a large study and provides relatively tight 95% confidence intervals especially for the intra-physician

**Table 7:** Mean standard deviation for imaging sites

**Imaging center**	**Anterior**	**Posterior**	**RightPost**	**Right**	**LeftPost**	**Left**	**Superior**	**Inferior**	**Volume**
**(IC)**	**(mm)**	**(mm)**	**(mm)**	**(mm)**	**(mm)**	**(mm)**	**(mm)**	**(mm)**	**(Cm**^**3**^**)**
**A**	1.6	*0.9*	1.8	1.8	1.7	1.5	1.9	2.3	6.6
**B**	**3.3**	*0.9*	1.6	1.5	1.5	1.2	2.7	2.5	7.7
**C**	1.8	*0.9*	1.3	1.5	1.6	1.2	2.0	2.3	6.7
**D**	2.4	**2.5**	**2.7**	1.4	**2.7**	1.6	2.0	2.9	**10.8**
**E**	*1.4*	1.4	2.2	2.0	2.4	2.0	1.7	2.3	6.4
**Home**	1.7	0.9	1.4	1.3	1.6	1.4	1.8	2.5	6.1
**Foreign**	2.3	1.6	2.1	1.7	2.1	1.6	2.1	2.5	8.1

**Bold** figures mean statistically significant larger and *italic* significantly smaller variation compared to other imaging centers (bonferroni correction p < 0.01) F-test. The two last rows compares the average standard deviation for delineations performed on images from physicians home center compared to images from a foreign center.

 variability, $\sigma_{z}^{\mathrm{var}}$. A common method for determination of confidence intervals for the true standard deviation *σ* is to make use of the relation between the ratio the of estimated, *s*, and true standard deviation and Chi^2^ distribution

(6) $$\frac{\mathrm{DF}.s^{2}}{\sigma^{2}}\sim \chi_{\mathrm{DF}}^{2}$$

Where *DF* is the degrees of freedom. For the intra-physician variation in the present study *DF* = (*N*_Q_ - 1)(*N*_P_ - 1). The confidence interval for $\sigma_{w}^{\mathrm{var}}$ can also be estimated using equation 6 (*DF* = *N*_Q_ - 1). but especially in cases when the $\sigma_{w}^{\mathrm{var}}\ll \sigma_{z}^{\mathrm{var}}$ this estimation will lead to an underestimation of the confidence interval. For the prostate, where the intra-physician and inter-physician variability was of the same magnitude, the use of equation 6 gives approximately the same results as simulation. For example in the inferior direction for the prostate, where the simulations and equation 6 gave equivalent results with 0.1 mm precision. But for the vesicles, where the intra-physician variability was larger compared to the inter-physician variability, the use of equation 6 under-estimates the confidence interval. For example the confidence interval for the inter-physician variability in the posterior direction was simulated to 2.1-0.6 mm, while equation 6 gave 1.9-0.7 mm. This can be understood considering the scenario with a very large intra-observer variation creating random variations between physicians, and making the inter-observer variation difficult to quantify. The reporting of confidence intervals is very important, especially when using small sample sizes and/or separation of variances into components.

**Figure 5:**
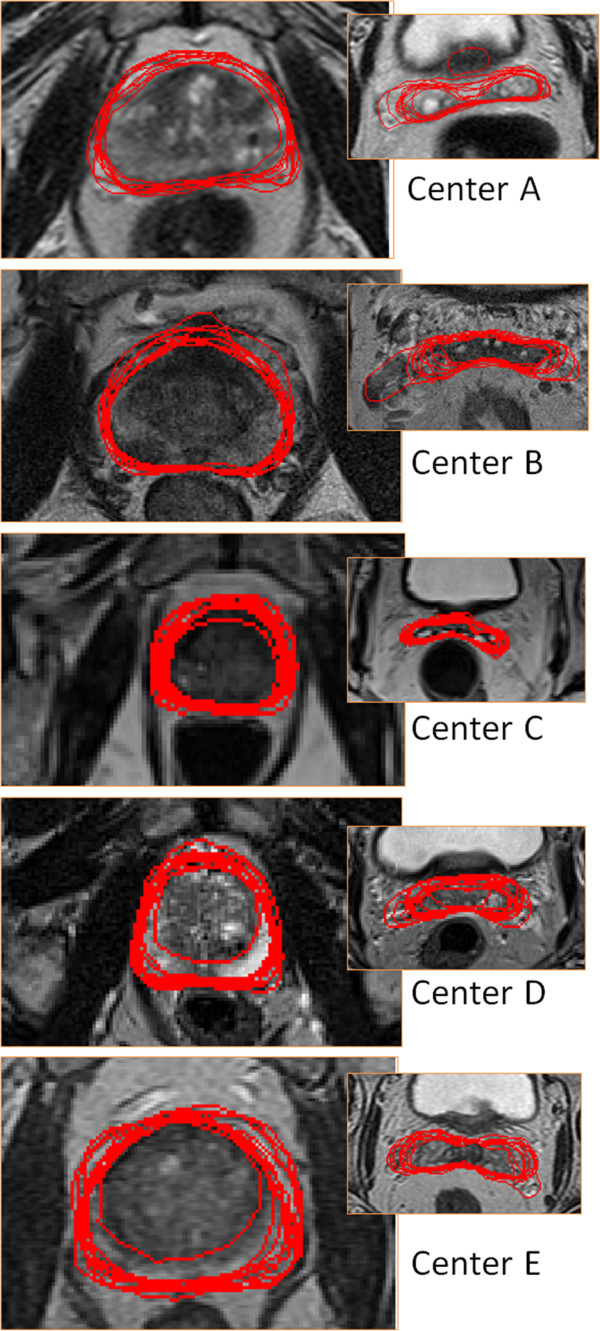
Examples of the delineations (prostate and seminal vesicles).

A clinical objective of the present work was to provide feedback to physicists optimizing the MR sequences at the different sites and to the physicians doing the delineations. For the physicians the feedback consisted of two parts. The images sets for the 25 patients were returned to each physician with their own delineations shown as a white structure set and the other 9 delineations shown as black structures. See Figure 5 for example of the delineation variability. The purpose of this feedback was to give an indication of their performance in relation to the others. In addition a one day workshop was organized where a selection of the patient cases were reviewed together with two radiologists specialized in MR examinations of the prostate and seminal vesicles. The outcome of this workshop was that the average delineation of the

**Table 8:** Mean standard deviation for imaging sites (vesicles)

**Imaging center**	**Anterior**	**Posterior**	**Right**	**Left**	**Superior**	**Inferior**	**Volume**
**(IC)**	**(mm)**	**(mm)**	**(mm)**	**(mm)**	**(mm)**	**(mm)**	**(cm**^**3**^**)**
**A**	3.2	*1.2*	*2.6*	3.0	2.3	*2.2*	*2.7*
**B**	3.0	3.4	3.1	4.2	3.8	3.6	5.2
**C**	4.0	*1.8*	*2.9*	2.9	2.9	2.9	*3.7*
**D**	**6.2**	**4.0**	5.6	**5.3**	4.0	**5.1**	**11.3**
**E**	2.8	3.4	3.6	3.4	2.7	3.9	*2.8*
**Home**	3.9	2.4	2.9	2.7	3.2	3.9	5.2
**Foreign**	4.0	3.1	5.0	4.1	3.2	3.6	6.2

**Bold** figures mean statistically significant larger and *italic* significantly smaller variation compared to other imaging centers (Bonferroni correction p < 0.01) F-test. The two last rows compares the average standard deviation for delineations performed on images from physicians home center compared to images from a foreign center.

 prostate from the 10 radiation oncologists was close to the opinion of the radiologists, while the delineations of the vesicles performed by the radiation oncologists tended to overestimate the extent of the seminal vesicles for some patients, especially in the anterior and right-left directions. The radiologists preferred the image quality provided by center B, followed by the image quality from center D. It is interesting to notice that the images from these sites were associated with the largest delineation variability. Our interpretation is that increased amount of information increases the scope for interpretation and hence the importance of training and experience. It also highlights the complexity of the optimization procedure and the importance of a well defined objective for the optimization. If the objective is to reduce the delineation variability of the prostate or the seminal vesicles it could be counter-productive to use sequences optimized to visualize pathology. Our opinion is that recommendations on specific sequence settings are difficult to make because of the different needs and possibilities at different centers. For example, if high quality diagnostic images are already available for a patient there is less need to acquire images optimized for pathology.

## Conclusion

The overall intra- and inter-physician variability for prostate and seminal vesicle delineations was determined for clinically used MR sequences optimized for target volume determination at 5 different radiotherapy centers in Europe. Large differences in variability were observed between different patients, but also between different MR sequences, even though all centers used T2-weighted spin-echo based sequences. The intra-physician variability was significantly larger for the seminal vesicles compared to the prostate, while the inter-physician variability was approximately the same.

## Competing interests

The author(s) declare that they have no competing interests.

## Authors’ contributions

TN, BZ and JJ conceived the study. All authors participated in the design of the study, data generation and data collection. TN and JJ drafted the manuscript. All authors read and approved the final manuscript.
